# *Clostridioides difficile* infection after extracorporeal membrane oxygenation support for acute myocardial infarction: a case report

**DOI:** 10.3389/fmed.2023.1333209

**Published:** 2023-12-22

**Authors:** Yanan Hu, Chang Hu, Jun Jiang, Jing Zhang, Yiming Li, Zhiyong Peng

**Affiliations:** ^1^Department of Critical Care Medicine, Zhongnan Hospital of Wuhan University, Wuhan, Hubei, China; ^2^Clinical Research Center of Hubei Critical Care Medicine, Wuhan, Hubei, China; ^3^Center of Critical Care Nephrology, Department of Critical Care Medicine, University of Pittsburgh School of Medicine, Pittsburgh, PA, United States

**Keywords:** *Clostridioides difficile*, *Clostridioides difficile* infection, fecal microbiota transplantation, extracorporeal membrane oxygenation, case report

## Abstract

**Introduction:**

Restored cardiopulmonary function is efficiently achieved by utilizing extracorporeal membrane oxygenation (ECMO). Nevertheless, the incidence of *Clostridioides difficile* infection (CDI) associated with ECMO is relatively uncommon.

**Case presentation:**

In this report, we present the case of a 59-year-old male with severe chest pain due to acute myocardial infarction, subsequently necessitating ECMO support. During the first day of hospitalization, pulmonary infections were observed, and piperacillin-tazobactam was prescribed for 7 days at low dosages. However, the patient developed severe diarrhea 4 days later. After ruling out common pathogens, we suspected the occurrence of CDI and performed genetic testing for *C. difficile* toxin, confirming our diagnosis. The prescription of vancomycin resulted in slight improvement, while fecal microbiota transplantation (FMT) proved to be more effective.

**Conclusion:**

In this case, temporary application of ECMO was applied, and the anti-infective treatment relied on the use of antibiotics at short-term, low-dose, and low CDI risk. Hence, the occurrence of CDI was considered an uncommon event, which may serve as a reference for future cases.

## Introduction

*Clostridioides difficile* infection (CDI) is a type of bacterial infection that primarily affects intestines. It arises from the disruption of the normal intestinal flora, which facilitates the colonization of *C. difficile* (1). Although CDI was traditionally considered a hospital-acquired infection, recent studies have indicated a significant proportion of community residents affected by *C. difficile* (2). Statistics show that approximately half a million individuals in the United States were infected with *C. difficile*, leading to a substantial annual cost of $4.8–6.3 billion for in-hospital management (3, 4). Several patient-related risk factors contribute to the risk of CDI, with antibiotics exposure being the most significant risk factor. The use of various antibiotics, particularly cephalosporins, clindamycin, fluoroquinolones, and carbapenems, is associated with the development of CDI. The risk of infection also increases with the number and duration of antibiotic applications. Furthermore, CDI is closely associated with advanced age, hospitalization, cancer, chronic kidney diseases, and the usage of immunosuppressants or gastrointestinal interventions like surgeries, nasal feeding, proton pump inhibitor (PPI), histamine-2 receptor antagonist (H_2_RA) (1, 5). The manifestation of CDI varies widely, ranging from asymptomatic carriage and mild to moderate diarrhea, to severe cases of fulminant colitis that can be fatal (6). Intensive Care Unit (ICU) patients, due to their critical and severe condition, often require concurrent antibiotics, nasogastric tube intubation, and intensive care, placing them at a higher risk of CDI. Therefore, it is crucial to emphasize universal prevention measures and carefully consider CDI as a potential cause when evaluating suspected symptoms.

Extracorporeal membrane oxygenation (ECMO) is a form of extracorporeal life support (ECLS) used for addressing circulatory or respiratory failure in patients. This technique utilizes a mechanical device that provides either short- or long-term extracorporeal life support. The most frequent conditions that warrant ECMO initiation are acute respiratory distress syndrome (ARDS) and cardiogenic shock (7). Despite its frequent utilization in the ICU, ECMO is a highly invasive procedure associated with multiple complications, including large vessel tears, hemorrhage, hemolysis, and infection. It is crucial to acknowledge that patients undergoing ECMO are typically critically ill, malnourished, or immunocompromised, which markedly increases the risk of infection (8). Stefano Biffi et al. (9) reported that lower respiratory tract infection was the most frequent type of infection in adults undergoing ECMO, with bloodstream infections, urinary tract infections, surgical site infections, and intestinal infections being less frequent. Additionally, Wang’s study (10) indicated that gram-negative pathogens, including *Acinetobacter baumannii* and *Klebsiella pneumoniae*, were the primary pathogens, while fungal pathogens like *Candida albicans* and *Candida glabrata* were also prevalent. In contrast, gram-positive pathogens were rarely observed. Based on clinical experience with ECMO, the incidence of *Clostridioides difficile* infection following ECMO support is uncommon among ICU patients. It is suspected that surgical interventions and intensive care associated with ECMO may promote the translocation of normal intestinal microbiota and increase the likelihood of *C. difficile* colonization.

Herein, this study reports on a patient suffered acute myocardial infarction and received ECMO treatment, who subsequently developed *C. difficile* infection. The clinical data will provide valuable insights for preventing and treating similar cases in the future.

## Case presentation

The patient was a 59-year-old male with a history of diabetes and hypertension. On November 9, 2021, at 4 pm, he experienced sudden chest pain and tightness without any apparent triggers, leading to his immediate transfer to the local hospital’s emergency department. An electrocardiogram (ECG) was performed, revealing an acute inferior myocardial infarction and a third-degree atrioventricular block. The patient was then received Alteplase at the standard dose. Shortly after, a follow-up ECG showed slight ST-segment resolution, but the patient had already entered a state of shock. To receive coronary stent implantation, he was immediately transferred to a higher-level hospital. However, during the surgery, the patient’s blood pressure and pulse oxygen saturation remained unstable. Temporary measures were taken, including the administration of norepinephrine (NE) at a rate of 160 ug/min, dopamine (DA) at 10 ug/min, and epinephrine at 20 ug/min. The patient was then admitted to the ICU at Zhongnan Hospital of Wuhan University, where he received tracheal intubations and an intra-aortic balloon pump (IABP). On the November 11 (Day 1), Veno-Arterial ECMO (VA-ECMO) support was provided to avoid deterioration.

The patient’s physical examination revealed body temperature of 36.5°C, heart rate of 72 bpm, SpO_2_ of 86%, respiration rate of 16 bpm while receiving endotracheal intubation and ventilator support using pressure control mode, with FiO_2_ set at 100%, frequency (f) set at 16 bmp, positive end-expiratory pressure (PEEP) at 12 cmH_2_O, and blood pressure reading of 144/99 mmHg (with NE injection at 80 ug/min). The patient’s bilateral pupil diameter was approximately 2.0 mm, with no icteric sclera and retained light reflexes. Cardiac sounds were low and unremarkable, and no other significant physical findings were noted. The laboratory results showed a white blood cell (WBC) count of 11.13 × 10^9^/L, C-reactive protein (CRP) level of 171.2 mg/L, procalcitonin (PCT) level of 46.17 ng/mL, brain natriuretic peptide (BNP) level of 334.8 pg./mL, creatine kinase (CK) level > 4,267 u/L, creatine kinase-MB (CK-MB) level of 823 u/L, myoglobin level > 1200.0 ng/mL, and high-sensitivity troponin I level > 50000.0 pg./mL. The arterial blood gas analysis reflected a pH of 7.21, PaO_2_ of 57.49 mmHg, PaCO_2_ of 50.55 mmHg, K^+^ of 6.04 mmol/L, Na^+^ of 138.39 mmol/L, HCO3^−^ of 19.8 mmol, and Lac of 4.28 mmol/L. Regarding the patient’s VA-ECMO settings, the speed was set at 7000 rpm/min, blood flow velocity at 3.3 L/min, gas flow velocity at 2 L/min, and fraction of inspired oxygen at 60%.

**Figure 1:**
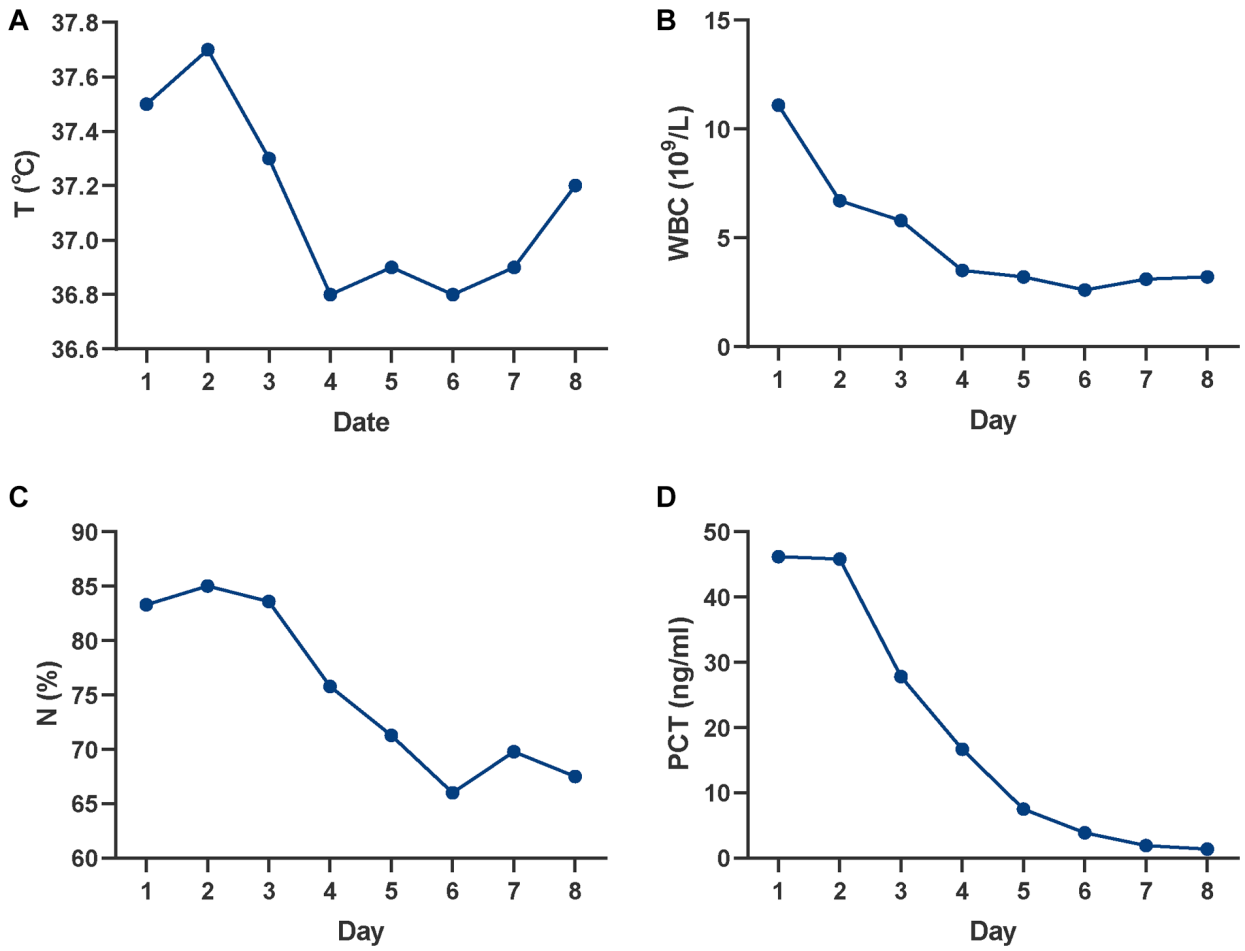
Changes in infectious indicators over time. **(A)** Changes in T; **(B)** Changes in WBC; **(C)** Changes in N%; **(D)** Changes in PCT. T, temperature; WBC, white blood cell; N%, neutrophil percentage; PCT, procalcitonin.

The patient initially suffered from acute myocardial infarction, despite attempts with medication and emergency surgery, these treatments proved ineffective. Therefore, VA-ECMO was promptly initiated to delay heart failure and provide additional time for cardiac function recovery. Fortunately, after 5 days of ECMO support, the patient’s general condition and heart function returned to normal, as indicated by stable vital signs and cardiac ultrasound (CUS) results. VA-ECMO was discontinued on the sixth day of hospitalization. Table 1 provides a record of the ECMO operating parameters and changes in left ventricular ejection fraction (LVEF). In addition to the primary condition, routine blood tests on day 1 showed abnormal indices: WBC: 11.1 × 10^9^/L, neutrophil percentage (N%): 83.3%, procalcitonin (PCT): 46.2 ng/mL, and interleukin-6 (IL-6): 660 pg./mL, along with a low-grade fever of 37.5°C. These findings suggested the occurrence of an infection, which was confirmed by chest X-ray and computed tomography (CT) showing pulmonary infections. As a result, piperacillin-tazobactam (iv. 4.5 g q8h) was prescribed for temporary control. Although clinical signs of infection were evident, specific bacteria were not detected in the sputum culture, blood culture, or bronchoalveolar lavage fluid (BLF). The infection was gradually improved with the prescribed medication (Figure 1).

**Table 1:** Detailed parameters of ECMO and the change in LVEF.

Date	ECMO parameters				LVEF (%)
	Speed (rpm/min)	Blood flow velocity (L/min)	Gas flow velocity (L/min)	FiO_2_ (%)	
Day 1	7,000	3.3	2	60	42
Day 2	7,300	3.2	3	80	25
Day 3	7,300	3.6	2	60	28
Day 4	5,900	3.1	2	60	32
Day 5	5,600	2	1	50	42
Day 6	Removed ECMO				55
Day 7	Removed IABP				54
Day 8	Took off NE				52

ECMO, extracorporeal membrane oxygenation; LVEF, left ventricular ejection fraction; IABP, intra-aortic balloon pump; NE, norepinephrine.

On Day 12, the patient’s condition deteriorated since he developed diarrhea. The diarrhea was characterized by yellow, water-like stools and the patient defected seven times that day, amounting to approximately 1,100 mL in total. The following day, the patient experienced eight bowel movements, with the volume increasing to 1,320 mL. Despite the prescription of antidiarrheal medications, including berberine and montmorillonite powder, the patient’s condition did not improve. Bacterial culture, biochemical identification, and susceptibility tests were performed on the fecal sample, but they did not yield any useful information. Considering the patient’s medical history of antibiotic administration during the first week of hospitalization, *Clostridioides difficile* was suspected as the likely cause of the persistent diarrhea. Genetic testing, using a real-time polymerase chain reaction assay for *C. difficile* toxin, confirmed that the patient tested positive for toxin B. Coupled with a WBC count exceeding 15 × 10^9^/L and creatinine (Cr) above 1.5 mg/dL, the patient was diagnosed with a severe CDI.

According to the ACG Clinical Guidelines (11), the patient was initially treated with vancomycin at a dosage of 125 mg every 6 h via a nasogastric tube. As a result, the patient’s stool frequency decreased to 3 times per day on Day 15, and then stabilized at 5–6 times per day with partially formed stool for the subsequent 4 days. Despite some improvement in diarrhea after 7 days of vancomycin treatment, the patient’s stool consistency did not fully return to a healthy state. Therefore, fecal microbiota transplantation (FMT) was proposed to prevent the progression of intestinal infections. Fecal samples were obtained from the Second Hospital Affiliated with Nanjing Medical University and were stored at a temperature of −20°C after being transported under low temperature conditions. The FMT infusion was administered on Day 20.

Before the operation, the samples were thawed in 37°C water for 1 h, and the patient was assisted to sit upright with intravenous metoclopramide (10 mg). Then, a nasal jejunal feeding tube was pre-placed, and the sample was infused through the tube. The whole procedure was completed within 3–5 min, and normal saline (5 mL) was used to flush the tube. The effect of the FMT was immediate, resulting in a significant reduction in stool frequency (3 times per day with a total volume of 330 mL on Day 21). In the following week, the patient experienced approximately 2–4 bowel movements per day, with a lower volume than before. Furthermore, genetic tests for *C. difficile* toxin conducted on both Day 24 and Day 28 yielded negative results, indicating a good outcome of FMT therapy for CDI. Detailed data on the development of CDI are presented in Table 2.

**Table 2:** Changes in fecal condition and relevant treatments.

Date	Fecal properties	Frequency (d^−1^)	Volume (ml)	Treatments
Day12	yellow, loose	7	1,100	Berberinum and Montmorillonite
Day13	yellow, loose	8	1,320	diagnosis of CDI
Day14	yellow, loose	6	1800	vancomycin 125 mg q6 h
Day15	yellow, loose	3	260	vancomycin 125 mg q6 h
Day16	yellow, loose	5	850	vancomycin 125 mg q6 h
Day17	yellow, loose	6	290	vancomycin 125 mg q6 h
Day18	yellow, partly formed	5	460	vancomycin 125 mg q6 h
Day19	yellow, partly formed	6	550	vancomycin 125 mg q6 h
Day20	yellow, partly formed	8	550	FMT
Day21	yellow, partly formed	4	330	–
Day22	yellow, formed	2	50	–
Day23	yellow, formed	4	240	–
Day24	yellow, formed	6	450	retest: tcd B (−)
Day28	yellow, formed	2	300	retest: tcd B (−)

CDI, *Clostridioides difficile* infection; FMT, fecal microbiota transplantation; tcd B, *Clostridioides difficile* toxin B.

**Figure 2:**
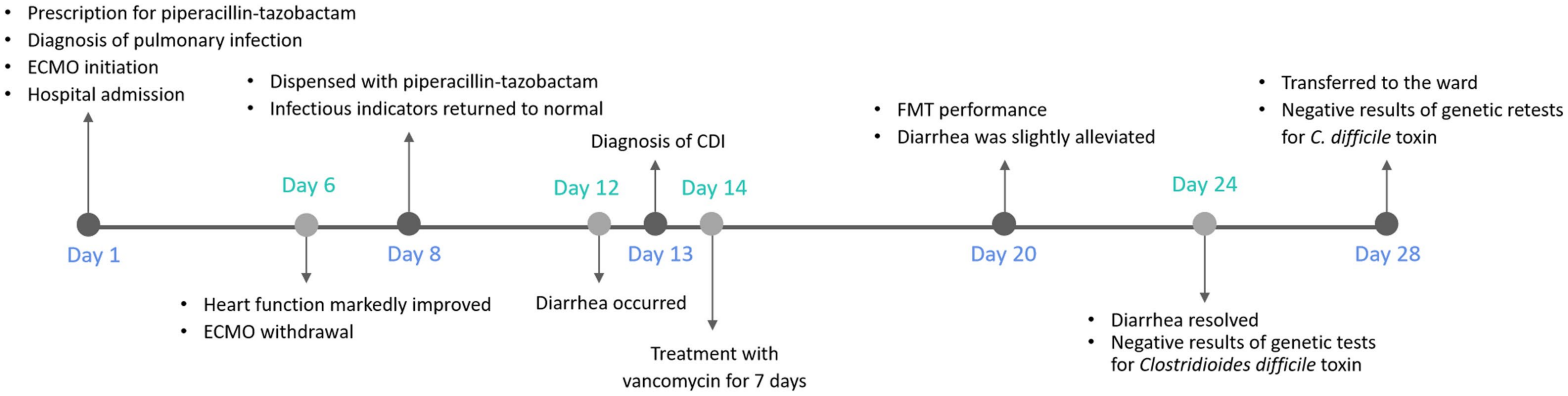
Diagnosis and treatment timeline of the patient. The timeline shows the major clinical events during the patient’s hospitalization. ECMO, extracorporeal membrane oxygenation; CDI, *Clostridioides difficile* infection; FMT, fecal microbiota transplantation.

After 28 days of ICU treatment, the patient regained consciousness, exhibited a good mood and demonstrated normal cardiac function, and regular bowel movements without any complaints of infections or intestinal discomfort. Comprehensive assessments led to the patient’s transfer from ICU back to the ward, where close monitoring was continued during the early phase of recovery. The whole process is illustrated in Figure 2.

## Discussion

ECMO is a crucial intervention for replacing cardiac and pulmonary functions in the short term. VA-ECMO is primarily used to manage heart failure, including cardiogenic shock following myocardial infarction, extracorporeal cardiopulmonary resuscitation (ECPR), and fulminant myocarditis (7). In this case study, the patient was admitted to the hospital due to acute myocardial infarction and initially received empiric treatment with thrombolytic drugs and IABP. However, the effectiveness of the treatment was limited, and the patient’s symptoms worsened. Consequently, ECMO was employed to provide more consummate support. Nonetheless, the duration of ECMO application increased, thereby increasing the likelihood of bacterial infection (12). Thus far, no reports have been published regarding *Clostridioides difficile* infection in patients undergoing ECMO support.

CDI is a leading cause of infectious diarrhea in medical institutions, with increasing incidence in the ICU (13, 14). This condition occurs from an imbalance in the normal gut flora, primarily due to the abuse of broad-spectrum antibacterial drugs and other contributing factors (15). Disturbances in the gut microbiota may lead to proliferation of *C. difficile* from both endogenous and exogenous sources. A meta-analysis comparing various antibiotic classes and their association with hospital-acquired CDI found that third-generation cephalosporins had the highest odds, followed by clindamycin, second-generation cephalosporins, and fourth-generation cephalosporins (16). In this report, the patient demonstrated infectious characteristics from the time of admission, including fever, increased leukocyte count, procalcitonin, neutrophils, and a diagnosis of pulmonary inflammation based on imaging. Piperacillin-tazobactam was used, resulting in the normalization of infection indicators, and antibiotics were discontinued after 7 days. Interestingly, numerous studies have shown a higher risk increase for CDI associated with broad-spectrum antimicrobials, such as cefepime and meropenem than with piperacillin-tazobactam (17–19). Kundrapu (20) explains that piperacillin-tazobactam has the potentially to combat the colonization of *C. difficile*, unlike cefepime and other cephalosporins, which supports a widely accepted hypothesis. Although the patient receiving antibiotics with a lower CDI risk and at a lower dosage for a shorter duration, it did not alter the patient’s CDI status. Several possible reasons were analyzed, including: (1) the spores of *C. difficile* were widely distributed in hospital settings, (2) the patient’s cardiogenic shock and 5-day ECMO support, rendering him more susceptible to multi-drug-resistant bacteria, like *C. difficile*, (3) the invasive facilities used in the ICU, such as mechanical ventilation and nasogastric tubes, which were major risk factors for CDI, (4) the disruption of gut flora balance by piperacillin-tazobactam as an antibiotic, leading to the colonization of various opportunistic pathogens, and (5) the patient’s concurrent conditions of hypoxic–ischemic encephalopathy and acute kidney injury during hospitalization, contributing to a decline in overall immune function.

The diagnosis of CDI necessitates a thorough evaluation of clinical symptoms and confirmatory tests. First, CDI should be suspected when patients present with acute diarrhea, defined as loose stools occurring three or more times in a 24-h period. The severity of symptoms can vary, with mild cases characterized by watery diarrhea and mild abdominal cramping or tenderness. Severe cases can present with extremely frequent voiding (10–15 times/day), intense abdominal pain, nausea, fever, and potentially life-threatening complications such as toxic megacolon, sepsis, and multiorgan failure. Second, the diagnosis of CDI requires positive genetic evidence or colonoscopic confirmation of pseudomembranous colitis (6, 21–23). According to the ACG clinical guidelines (11), a WBC count greater than 15 × 10^9^/L and a serum Cr level greater than 1.5 mg/dL classify the patient as having severe CDI.

Regarding the treatment of CDI, it is crucial to evaluate the discontinuation of antibiotic therapy for all patients diagnosed with CDI, unless it hampers the recovery of other conditions. Vancomycin is the preferred treatment for mild to severe CDI, with evidence supporting the use of fidaxomicin as an alternative. Metronidazole is suggested solely for the first episode of mild or moderate CDI if vancomycin or fidaxomicin are not available (24, 25). In this report, the patient was diagnosed with severe CDI and received vancomycin, which proved ineffective. The lack of effectiveness against the infection may be attributed to common antibiotic resistance.

CDI is rooted in gut-induced dysbiosis, leading to extensive research on microbial therapies. FMT is an advanced approach that restores the complete spectrum of microorganisms constituting normal colonic flora by transferring the colonic microbiome from a healthy individual. When standard medical therapy for severe CDI is ineffective, FMT should be considered a treatment option (26, 27). Serious adverse events associated with FMT are rare, and gastrointestinal symptoms such as nausea, belching, or bloating are the most commonly reported issues, particularly when FMT is administered through the upper gastrointestinal route (28, 29). The risk of infection transmission, such as enteropathogenic *Escherichia coli* (EPEC) or Shiga toxin-producing *Escherichia coli* (STEC), can be mitigated through careful donor selection and screening. Nevertheless, the acceptability of this application method to patients and the safety of transportation during the coronavirus disease 2019 (COVID-19) pandemic should be taken into account (30–32). In this report, FMT was used to treat CDI after unsatisfactory outcomes with vancomycin, resulting in significant declines in stool frequency and volume, as well as the absence of the toxin B gene detected in two subsequent tests conducted a few days later.

The prevention strategies for CDI hold paramount importance, with the primary approach being the restriction of antibiotic use, particularly high-risk antibiotics. In case where elderly individuals (aged 65 years or older), patients with a history of healthcare exposure or other inevitable risk factors for CDI, it is crucial to minimize the duration of antibiotic administrations as much as possible (33). Second, decontamination of hospital environments is imperative to prevent the transmission of CDI, as the spores of *C. difficile* are largely resistant and actively released from infected patients (34). Alongside ensuring high-quality disinfection and sterilization in hospitals, particular attention should be given to hand hygiene. Healthcare personnel in close proximity to patients must adhere to recommended procedures for thorough hand disinfection, using running water (35). It is worth noting that, at present, there is insufficient evidence to recommend any probiotics for the primary or secondary prevention of CDI in most patients (11).

## Conclusion

In the ICU, ECMO stands as an easily accessible medical advancement. However, it is important to acknowledge that patients requiring ECMO support often suffer from critical and emergent illnesses, which increase their likelihood of antibiotic use, complications, and immune system impairment. Under these conditions, the intestinal flora becomes particularly vulnerable to dysregulation, potentially leading to the development of CDI. Therefore, clinicians should prioritize the preservation and enhancement of gastrointestinal function, recognizing the paramount significance of implementing all reasonable precautions and treatments to positively influence therapy, prognosis, and healthcare costs. Both healthcare professionals and patients share a common goal of efficiently combatting the risk of CDI.

## Data availability statement

The original contributions presented in the study are included in the article/supplementary material, further inquiries can be directed to the corresponding authors.

## Ethics statement

Written informed consent was obtained from the individual(s) for the publication of any potentially identifiable images or data included in this article.

## Author contributions

YH: Conceptualization, Investigation, Writing – original draft. CH: Methodology, Supervision, Writing – original draft. JJ: Project administration, Writing – original draft. JZ: Resources. YL: Formal analysis, Supervision, Writing – review & editing. ZP: Funding acquisition, Resources, Writing – review & editing.

## Figures and Tables

**Table:** 

ECMO	extracorporeal membrane oxygenation
CDI	*Clostridioides difficile* infection
PPI	proton pump inhibitor
H_2_RA	histamine-2 receptor antagonist
ICU	Intensive Care Unit
ECLS	extracorporeal life support
ARDS	acute respiratory distress syndrome
ECG	electrocardiogram
NE	norepinephrine
DA	dopamine
IABP	intra-aortic balloon pump
VA-ECMO	Veno-Arterial ECMO
PEEP	positive end-expiratory pressure
WBC	white blood cell
CRP	reactive protein
PCT	procalcitonin
BNP	brain natriuretic peptide
CK	creatine kinase
CK-MB	creatine kinase-MB
CUS	cardiac ultrasound
LVEF	left ventricular ejection fraction
N%	neutrophil percentage
PCT	procalcitonin
IL-6	interleukin-6
CT	computed tomography
BLF	bronchoalveolar lavage fluid
Cr	creatinine
FMT	fecal microbiota transplantation
ECPR	extracorporeal cardiopulmonary resuscitation
EPEC	enteropathogenic *Escherichia coli*
STEC	Shiga toxin-producing *Escherichia coli*
COVID-19	the coronavirus disease 2019
